# A Meta-Analysis of the Relationship Between *RARβ* Gene Promoter Methylation and Non-Small Cell Lung Cancer

**DOI:** 10.1371/journal.pone.0096163

**Published:** 2014-05-05

**Authors:** Feng Hua, Nianzhen Fang, Xuebing Li, Siwei Zhu, Weisan Zhang, Jundong Gu

**Affiliations:** 1 Department of surgery oncology, Shandong cancer hospital, Jinan, China; 2 Tianjin Medical University General Hospital, Tianjin Lung Cancer Institute, Tianjin Key Laboratory of Lung Cancer Metastasis and Tumor Microenvironment, Tianjin, China; 3 Department of Oncology, Tianjin Union Medical Center, Tianjin, China; 4 Department of Geriatrics, Tianjin Medical University General Hospital, Tianjin, China; Duke Cancer Institute, United States of America

## Abstract

**Background:**

Hypermethylation of CpG islands in tumor suppressor gene plays an important role in carcinogenesis. Many studies have demonstrated that hypermethylation in promoter region of *RARβ* gene could be found with high prevalence in tumor tissue and autologous controls such as corresponding non-tumor lung tissue, sputum and plasma of the NSCLC patients. But with the small number subjects included in the individual studie, the statistical power is limited. Accordingly, we performed this meta-analysis to further asses the relationship of methylation prevalence between the cancer tissue and atuologous controls (corresponding non-tumor lung tissue, sputum and plasma).

**Methods:**

The published articles about *RARβ* gene promoter hypermethyltion were identified using a systematic search strategy in PubMed, EMBASE and CNKI databases. The pooled odds ratio (OR) of *RARβ* promoter methylation in lung cancer tissue versus autologous controls were calculated.

**Results:**

Finally, eleven articles, including 1347 tumor tissue samples and 1137 autologous controls were included in this meta-analysis. The pooled odds ratio of *RARβ* promoter methylation in cancer tissue was 3.60 (95%CI: 2.46–5.27) compared to autologous controls with random-effect model. Strong and significant correlation between tumor tissue and autologous controls of *RARβ* gene promoter hypermethylation prevalence across studies (Correlation coefficient 0.53) was found.

**Conclusion:**

*RARβ* promoter methylation may play an important role in carcinogenesis of the NSCLC. With significant methylation prevalence correlation between tumor tissue and autologous of this gene, methylation detection may be a potential method for searching biomarker for NSCLC.

## Introduction

Lung cancer, accounting for 100 million deaths and 120 million incidence per year world-wide, is the leading cause of cancer related death[1]. Generally, lung cancer is divided in to non-small cell lung cancer (NSCLC) and small-cell lung cancer according to the pathology. The prognosis of NSCLC, accounting for 80–85% of the lung cancer, is poor for lack of effective early diagnosis methods[2]. Recently, CpG island methylation of tumor suppressor genes promoters have been found to be important for carcinogenesis in many kinds of carcinomas including NSCLC[3]. The DNA methylation is a biochemical process involving the addition of a methyl group to thecytosine or adenine DNA nucleotides. DNA methylation may affect the transcription of the tumor suppressor genes in two ways. Firstly, the methylated DNA may physically impede the binding of transcriptional proteins to the gene itsel[4]. Secondly, may be with more important, methylated DNA may be bound by proteins known as methyl-CpG-binding domain proteins (MBDs)[5]. MBD proteins then recruit additional proteins to the locus, such as histone deacetylases and other chromatin remodeling proteins that can modify histones, thereby forming compact, inactive chromatin, termed heterochromatin[6].

*RARβ* gene is a typical tumor suppressor gene encoding retinoic acid receptor beta, a member of the thyroid-steroid hormone receptor subfamily, which belongs to nuclear receptor superfamily. The protein encoded by this gene could bind retinoic acid, the biologically active form of vitamin A which mediates cellular signallings in embryonic morphogenesis, cell growth and differentiation[7]. Recent studies demonstrated that hypermethylation in promoter region of *RARβ* gene could be found with high prevalence in tumor tissue and autologous controls such as corresponding non-tumor lung tissues (CNTLT), sputum and plasma of the NSCLC patients. But with the small number subjects included in the individual studies, the statistical power is limited. Thus, a meta-analysis about the relationship of *RARβ* promoter methylation between lung cancer tissue and autologous controls was done in order to better identify the correlation of methylation status between cancer tissue and autologous samples.

## Methods

### Studies identification

**Figure 1:**
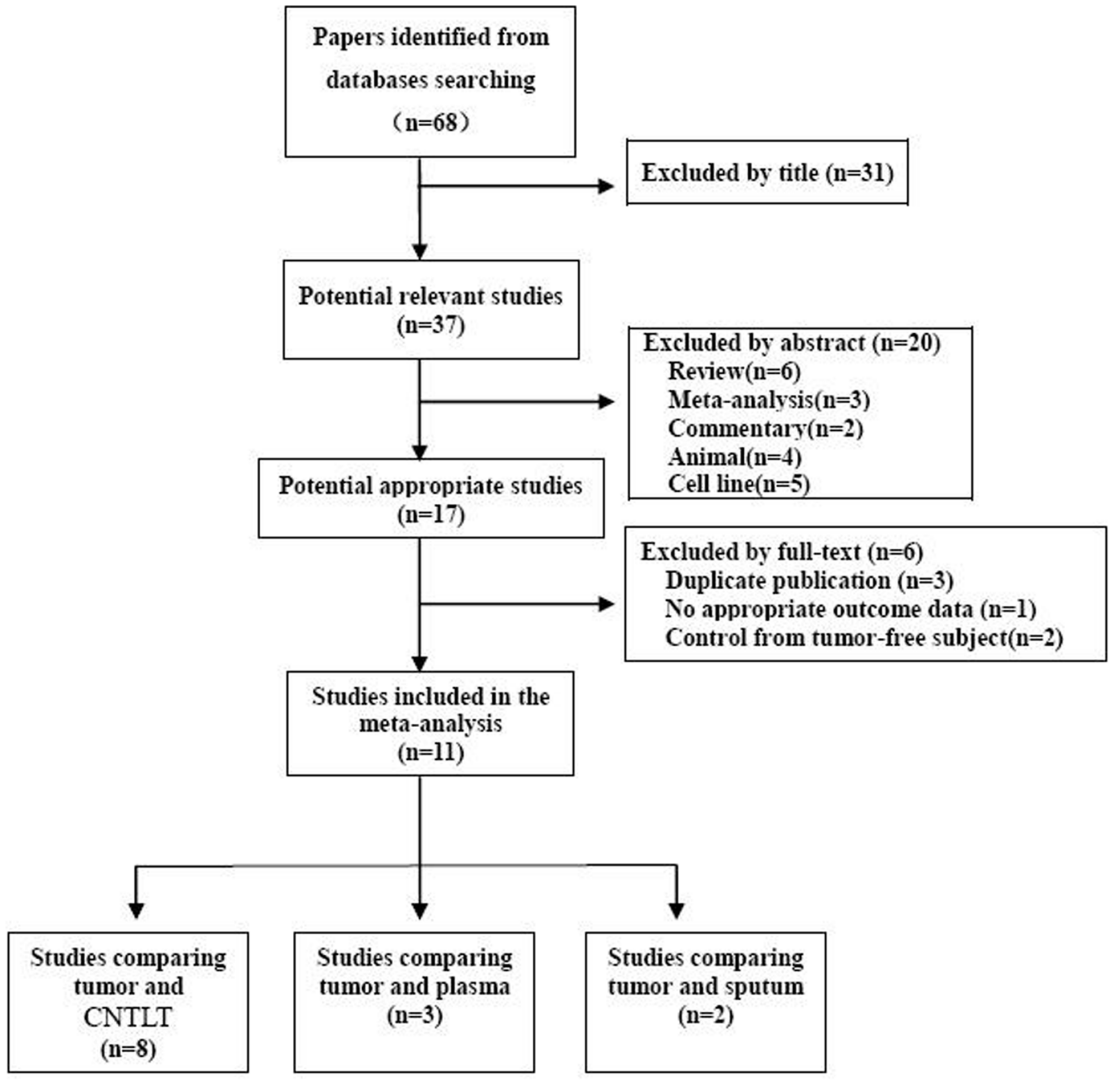
Flowchart of the literature search strategy (Data from some studies was used more than once).

The selection procedure of the relevant articles was demonstrated in figure 1. Papers about *RARβ* gene promoter CpG island hypermethylation in NSCLC, published before February 2013, were identified by searching Pubmed, EMBASE and CNKI databases. The “Non-small cell lung carcinoma/NSCLC” “methylaiton/hypermethlation” and “*RARβ/RAR-beta*” were used as the MeSH and free text word when searching the databases. The inclusion criteria: non-small cell lung cancer patients with histology or cytology conformation; The methylation was detected by methylation-specifec PCR (MSP) or quantitative MSP (q-MSP); The methylation rate in the cancer tissue and autologous controls would be drawn in the original studies included in this meta-analysis. All potentially relevant articles were assessed in full-text paper and all references of included articles were scanned for additional analysis according to the Cochrane handbook for systematic review[8].

### Data extraction

Information about the general characteristics and methylation prevalence of the original included studies were retrieved. For general characteristics: The first author, year of publication, journals, race of the included subjects, mean or median age of the included patients, methylation detection methods were all recorded. For methylation prevalence in cancer tissue and autologous controls: the *RARβ* promoter methylation rate or number of methylated (M) and unmethylated (U) samples in cancer tissue and autologous controls were all retrieved from the included studies. The methylation prevalence (MP) was calculated according to the formula PM = M/(M+U)×100%. All of the information details were extracted by two reviewers (FH and NZF) and then checked by the third reviewer (JDG).

### Statistical analysis

The odds ratio (OR) for each individual study was calculated by the formula OR = (a/b)/(c/d). (a =  number of methylated samples in tumor tissue b = number of unmethylated samples in tumor tissue; c = number of methylated in control tissue and d = number of unmethylated in control tissue.) The OR and its 95% confidence intervals (CI) of *RARβ* promoter hypermethylation in cancer tissue compared to autologous controls (CNTLT, sputum and plasma) for the included studies was pooled by STATA/SE 11.0(StataCorp LP, http://www.stata.com)statistic software. Statistical heterogeneity among articles included in this meta-analysis was evaluated by I^2^ [9].Without significant heterogeneity (I^2^>50%), the fixed-effect method was used to pool the data. Inversely, with significant heterogeneity among studies, random-effect method (Dersimonian-Laird method) was taken to pool the OR. The methylation prevalence correlation between cancer tissue and autologous clinical control (CNTLT, sputum and plasma) was evaluated by Spearman's rank correlation test. And the publication bias was assessed by Begg's funnel plot and Egger's test [10].

## Results

### General characteristics of included studies

Finally, eleven articles[11]–[21], including 1347 tumor tissue samples and 1137 autologous controls, were included in this meta-analysis.(Figure 1). Of the 11 articles included in this meta-analysis, 6 were conducted in Chinese mainland, 2 were in Taiwan, 2 in USA and 1 in Italy. The median methylation prevalence of *RARβ* gene promoter was 47.56% and 17.50% in cancer tissue and autologous controls, respectively (table S1). The general characteristics of the included articles were showed in table1.

**Table 1:** General characteristics of included studies.

Author	Year	Location	Age(y)	Gender M/F	Sample size (n)		Method	Control type
					T	C		
Zhao[21]	2011	China	59.4±9.6	68/12	80	80	MSP	CNTLT
Hu[20]	2011	China	59.6±9.3	102/18	120	120	MSP	CNTLT
Hu[20]	2011	China	59.6±9.3	102/18	120	120	MSP	Plasma
Zhang[12]	2011	China	61.0	162/38	200	200	MSP	CNTLT
Song[11]	2011	China	59(35–80)	NA	78	78	MSP	CNTLT
Zhang[19]	2011	China	59(median)	58/20	78	110	MSP	Plasma
Feng[13]	2008	USA	NA	24/25	49	49	q-MSP	CNTLT
Hsu[14]	2007	Taiwan	69(median)	45/18	63	63	MSP	CNTLT
Hsu[14]	2007	Taiwan	69(median)	45/18	63	63	MSP	Plasma
Hsu[15]	2007	Taiwan	NA	NA	82	82	MSP	Sputum
Cirincione[16]	2006	Italy	NA	NA	29	18	Msp	Sputum
Yang[18]	2005	China	56±11	34/15	49	49	MSP	CNTLT
Toyooka[17]	2003	USA	NA	355/159	514	84	MSP	CNTLT

M = male; F = female; T = tumor; C = control; CNTLT =  Corresponding non-tumor lung tissues; NA = not aviable.

### Pooled results of this meta-analysis

**Figure 2:**
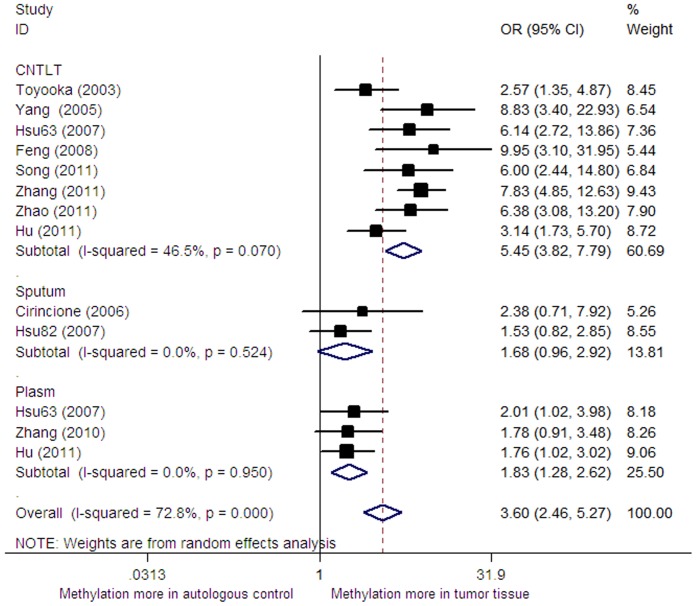
Forest plot of pooled OR for *RARβ* gene promoter methylation in cancer tissue versus autologous controls.

Significant heterogeneity was found across the included studies (I^2^ = 72.8%). Accordingly, the random-effect model was taken in pooling the OR. In this meta-analysis, the pooled odds ratio of *RARβ* promoter methylation in cancer tissue was 3.60 (95%CI: 2.46–5.27, p = 0.000) versus autologous controls with random-effect model (Figure 2). The sensitivity analysis was also done by omitting a single study under the random-effect model. But no significant changes of OR and it 95%CI was found in the sensitivity analysis which demonstrated that the pooled odds ratio was not sensitive to a single study.

### Subgroup analysis

With significant heterogeneity across the included studies (I^2^ = 72.8%), the subgroup analysis of this meta-analysis was performed according to race (East-asia, Caucasian) and control types (corresponding non-tumor lung tissues, sputum and plasma). In the subgroup analysis, the significant odds of the *RARβ* promoter methylation in tumor tissue was only changed when comparing to sputum (OR = 1.33, 95%CI: 0.98–1.80, *P* = 0.120) and in subjects with Caucasian ethnicity (OR = 2.38, 95%CI: 0.71–7.92, *P* = 0.159, Table 2). However, the conclusion of the sputum control and Caucasian ethnicity subgroup should be interpreted with caution as only a two and one articles with small subjects were included in these subgroups analysis.

**Table 2:** Subgroup analysis.

Subgroup	NSCLC		Control		OR	95%CI	p
	M+	Total	M+	Total			
Race							
ast-asian	459	946	209	986	3.59	2.30–5.61	0.000
aucasus	19	29	8	18	2.38	0.71–7.92	
Control type							
NTLT	491	1160	116	735	5.45	3.82–7.79	0.000
lasma	106	268	78	302	1.83	1.28–2.62	0.001
putum	58	110	39	100	1.68	0.96–2.92	0.067

### Methylation prevalence correlation

**Figure 3:**
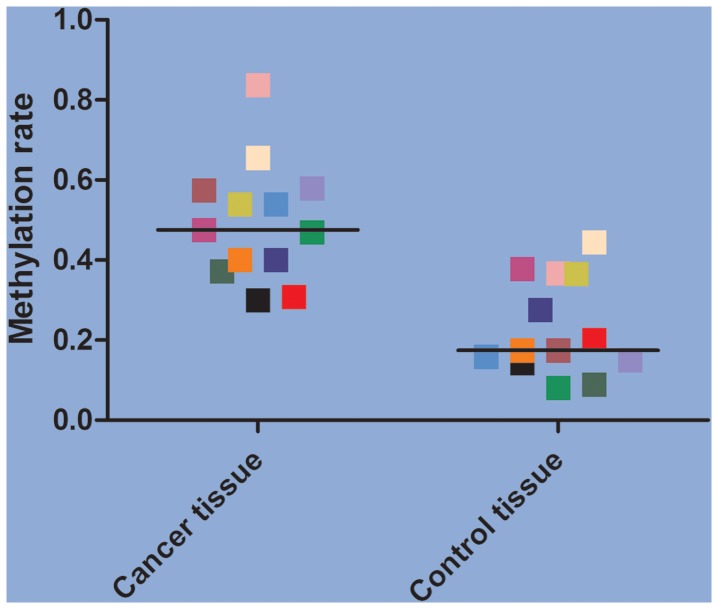
The distribution of methylation rate for each included study (the paired tissue were in the same colour).

**Figure 4:**
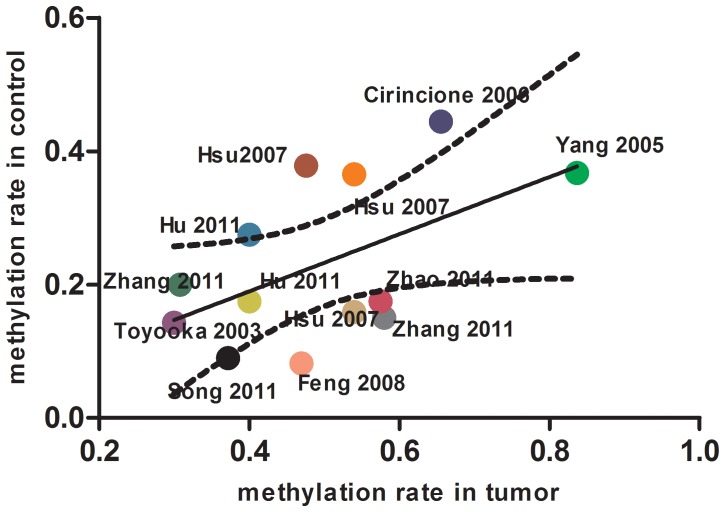
Correlation of *RARβ* gene promoter methylation between tumor tissue and autolougs controls.

The methylation prevalence was much higher in tumor tissue (meidan methylation prevalence 47.56%) compared to autologous controls (meidan methylation prevalence 17.50%) of *RARβ* gene (Figure 3). And we also found significant *RARβ* gene promoter hypermethylation prevalence correlation between tumor tissue and autologous controls among the included studies (Correlation coefficient 0.53) (Figure 4). It means that the more methylation percentage in tumor tissue and the more methylation percentage in autologous controls.

### Publication bias

**Figure 5:**
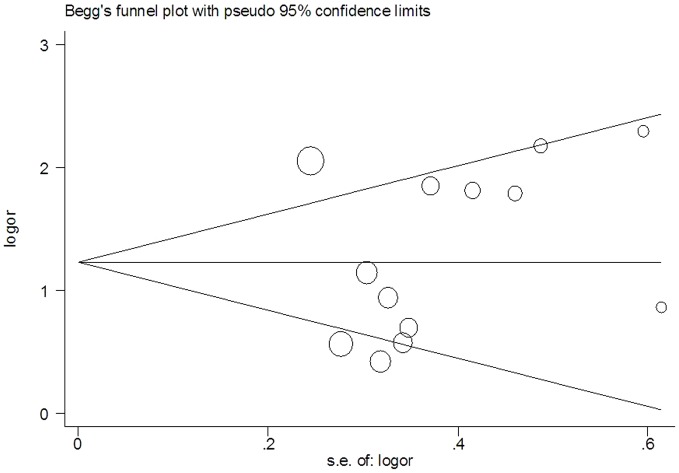
Begg's funnel plot for assessment of publication bias.

No statistical significant publication bias was detected by Begger's funnel plot and Egger's tests (t = 0.70, p = 0.500) (Figure 5).

## Discussion

Lung cancer is leading cause of cancer related mortality world-wide with 100 million deaths and 120 million diagnoses in year 2009[22]. Without effective early diagnosis method, the prognosis of NSCLC is relative poor with a 5-year survival rate of less than 15%[2]. CpG island methylation of tumour suppressor genes promoters have been found to be important for carcinogenesis and early events in many kinds of carcinomas including NSCLC. *RARβ* gene is a typical anti-tumor gene encoding retinoic acid receptor beta, a member of the thyroid-steroid hormone receptor subfamily of nuclear receptor superfamily. The protein encoded by this gene could bind retinoic acid, the biologically active form of vitamin A which mediates cellular signallings in embryonic morphogenesis, cell growth and differentiation. This gene was always been silence by its promoter DNA hypermethylation on its promoter region which can physically impede the binding of transcriptional proteins to the gene itself.

The relationship between cancer tissue and autologous controls such as CNTLT, plasma and sputum for this gene was not clear with small subjects in the individual studies. So, we performed this meta-analysis, which can strength the statistical power to quantify the hypermethylation-disease association by pooling data from open published articles. In this meta-analysis, we finally included eleven articles with 1347 tumor tissue samples and 1137 autologous controls to pool the odds ratio of methylation prevelance. The pooled odds ratio of *RARβ* promoter methylation in cancer tissue was 3.60 (95%CI: 2.46–5.27) compared to autologous controls with random-effect model. The subgroup analysis of this meta-analysis was performed according to race (East-asian, Caucasian) and control types. In the subgroup analysis, the significant odds of the *RARβ* promoter methylation in tumor tissue was only changed when comparing to sputum (p = 0.120) and in subjects with Caucasian ethnicity (p = 0.159). However, the conclusion of the sputum control and Caucasian ethnicity subgroup should be interpreted with caution as only a two and one articles with small subjects were included in these subgroup analyses. We also found strong and significant correlation between tumor tissue and autologous controls of *RARβ* gene promoter hypermethylation prevalence across studies (r = 0.53). The correlation showed that, the higher methylation prevalence in plasma/sputum samples, the higher methylation prevalence could be found in cancer tissue in patients with NSCLC. And this indicated that detection methylation status in plasma or sputum could be a useful assay for diagnosis of NSCLC without or with mirror invasion. Nevertheless, in our meta-analysis, all of samples (tumor tissue, non-tumor lung tissue sputum and plasma) are all come from the patients with confirmed lung cancer. There was no relative healthy person controls. So, the lung cancer screening sensitivity and specificity was not possible to calculate according to the Bayes theorem.

Some limitations of this meta-analysis are need for further consideration. First, significant heterogeneity was found in this meta-analysis (I^2^ = 72.8%), which could decrease the statistical power of this study. Second, the co-variate methylaiton status of different tumor suppressor genes may also be linked and interact with each other, suggesting methylation analysis of a single gene may be not enough[23].

In conclusion, this meta-analysis showed *RARβ* promoter methylation was much higher in tumor tissue compared to autologous controls, which indicates promoter hypermethylation of tumor suppressor gene may play an important role in carcinogenesis of the NSCLC. With significant methylation prevalence correlation, methylation detection may be a potential stragegy for searching biomarker for NSCLC [24].

## Supporting Information

Table S1 The methylation percentage in each included study
(DOC)

Checklist S1 Prisma checklist.
(DOC)
