# Supplementary material for: A Meta-Analysis of the Relationship Between RARβ Gene Promoter Methylation and Non-Small Cell Lung Cancer
Source: PLoS One. 2014 May 5;9(5):e96163. doi: 10.1371/journal.pone.0096163 (PMC4010458; doi:10.1371/journal.pone.0096163)
Supplement: Table S1 — The methylation percentage in each included study (DOC) [file pone.0096163.s001.doc]

| **Author** | **Year** | **Tumor** | | |  | **Control** | |  |
| --- | --- | --- | --- | --- | --- | --- | --- | --- |
|  |  | **M** | **U** | P |  | **M** | **U** | P |
| Zhao^21^ | 2011 | 46 | 34 | 0.58 |  | 14 | 66 | 0.18 |
| Hu^20^ | 2011 | 47 | 72 | 0.39 |  | 21 | 99 | 0.18 |
| Hu^20^ | 2011 | 48 | 72 | 0.40 |  | 33 | 87 | 0.28 |
| Zhang^12^ | 2011 | 116 | 84 | 0.58 |  | 30 | 170 | 0.15 |
| Song^11^ | 2011 | 29 | 49 | 0.37 |  | 7 | 71 | 0.09 |
| Zhang^19^ | 2011 | 24 | 54 | 0.31 |  | 22 | 88 | 0.20 |
| Feng^13^ | 2008 | 23 | 26 | 0.47 |  | 4 | 45 | 0.08 |
| Hsu^14^ | 2007 | 34 | 36 | 0.49 |  | 10 | 65 | 0.13 |
| Hsu^14^ | 2007 | 34 | 36 | 0.49 |  | 23 | 49 | 0.32 |
| Hsu^15^ | 2007 | 39 | 42 | 0.48 |  | 31 | 51 | 0.38 |
| Cirincione^16^ | 2006 | 19 | 10 | 0.66 |  | 8 | 10 | 0.44 |
| Yang^18^ | 2005 | 41 | 8 | 0.84 |  | 18 | 31 | 0.37 |
| Toyooka^17^ | 2003 | 154 | 360 | 0.30 |  | 12 | 72 | 0.14 |

Supplementary Table 1. The methylation percentage in each included study

M=methylated; U=unmethylated ; P=methylation percentage
